# NBS-Encoding Genes in *Brassica napus* Evolved Rapidly After Allopolyploidization and Co-localize With Known Disease Resistance Loci

**DOI:** 10.3389/fpls.2019.00026

**Published:** 2019-01-30

**Authors:** Ying Fu, Yaofeng Zhang, Annaliese S. Mason, Baogang Lin, Dongqing Zhang, Huasheng Yu, Donghui Fu

**Affiliations:** ^1^Institute of Crop and Nuclear Technology Utilization, Zhejiang Academy of Agricultural Sciences, Hangzhou, China; ^2^Department of Plant Breeding, IFZ Research Centre for Biosystems, Land Use and Nutrition, Justus Liebig University Giessen, Giessen, Germany; ^3^Key Laboratory of Crop Physiology, Ecology and Genetic Breeding, Ministry of Education, Agronomy College, Jiangxi Agricultural University, Nanchang, China

**Keywords:** nucleotide-binding site (NBS), disease resistance, *Brassica napus*, *Brassica oleracea*, *Brassica rapa*

## Abstract

Genes containing nucleotide-binding sites (NBS) play an important role in pathogen resistance in plants. However, the evolutionary fate of NBS-encoding genes after formation of allotetraploid *Brassica napus* (A_n_A_n_C_n_C_n_, 2n = 38) is still unknown. We performed a genome-wide comparison of putatively functional NBS-encoding genes in *B. napus* and its progenitor species *Brassica rapa* (A_r_A_r_, 2n = 20) and *Brassica oleracea* (C_o_C_o_, 2n = 18), identifying 464, 202, and 146 putatively functional NBS-encoding genes respectively, with genes unevenly distributed in several clusters. The A_n_-subgenome of *B. napus* possessed similar numbers of NBS-encoding genes (191 genes) to the A_r_ genome of *B. rapa* (202 genes) and similar clustering patterns. However, the C_n_ genome of *B. napus* had many more genes (273) than the *B. oleracea* C_o_ genome (146), with different clustering trends. Only 97 NBS-encoding genes (66.4%) in *B. oleracea* were homologous with NBS-encoding genes in *B. napus*, while 176 NBS-encoding genes (87.1%) were homologous between *B. rapa* and *B. napus*. These results suggest a greater diversification of NBS-encoding genes in the C genome may have occurred after formation of *B. napus.* Although most NBS-encoding genes in *B. napus* appeared to derive from the progenitors, the birth and death of several NBS-encoding genes was also putatively mediated by non-homologous recombination. The Ka/Ks values of most homologous pairs between *B. napus* and the progenitor species were less than 1, suggesting purifying selection during *B. napus* evolution. The majority of NBS-encoding genes (60% in all species) showed higher expression levels in root tissue (out of root, leaf, stem, seed and flower tissue types). Comparative analysis of NBS-encoding genes with mapped resistance QTL against three major diseases of *B. napus* (blackleg, clubroot and *Sclerotinia* stem rot) found 204 NBS-encoding genes in *B. napus* located within 71 resistance QTL intervals. The majority of NBS-encoding genes were co-located with resistance QTLs against a single disease, while 47 genes were co-located with QTLs against two diseases and 3 genes were co-located with QTLs against all three. Our results revealed significant variation as well as interesting evolutionary trajectories of NBS-encoding genes in the different *Brassica* subgenomes, while co-localization of NBS-encoding genes and resistance QTL may facilitate resistance breeding in oilseed rape.

## Introduction

Plants are surrounded by a vast array of invaders, such as viruses, bacteria, fungi, nematodes and pests, many of which can cause diseases (Dangl and Jones, 2001; Meyers et al., 2003; McHale et al., 2006). In order to cope with invader attacks, plants have evolved sophisticated immune mechanisms to protect themselves against their natural enemies. The best known are the vast numbers of resistance genes in plants, which play a central role in recognizing effectors from pathogens and in triggering downstream signaling during plant responses to pathogen invasions (Liu et al., 2007; Yang et al., 2013).

Genes containing a nucleotide-binding site (NBS), namely NBS-encoding genes, constitute one of the largest plant resistance gene families (∼80%) (Meyers et al., 2003; Liu et al., 2007; Yang et al., 2013). The NBS domain was found to bind and hydrolyze ATP or GTP, and primarily functions as a signal transduction switch following pathogen recognition (Dangl and Jones, 2001; Martin et al., 2003). NBS-encoding genes typically comprise three principal domains, with the NBS domain in the middle region, flanked by a leucine-rich repeat (LRR) domain at the C-terminus, and by a toll/interleukin-1 receptor (TIR) or coiled-coil (CC) at the N-terminus (Cannon et al., 2002; Meyers et al., 2003; Shao et al., 2014; Zhang et al., 2016). The central NBS domain encodes several motifs consisting of 10–30 amino acids (aa), and is typically highly conserved (Meyers et al., 1999; Yue et al., 2012), whereas the C-terminal LRR domain exhibits high diversity and has been associated with pathogen recognition (Kobe and Deisenhofer, 1995; Leister and Katagiri, 2000; Dangl and Jones, 2001). According to the presence or absence of the N-terminal TIR domain, NBS-encoding genes are further classified into TIR-NBS-LRR (TNL) or TIR-NBS (TN) genes and non-TIR-NBS-LRR (non-TNL) or (non-TN) genes (Meyers et al., 1999). Based on the presence of the CC or other domains at the N-terminus, non-TNL and non-TN genes can be further divided into CC-NBS-LRR (CNL) or CC-NBS (CN) genes and X-NBS-LRR (XNL) or X-NBS (XN) genes (Dangl et al., 2001).

With the availability of genomic data for an increasing number of species, a set of NBS-encoding genes has been identified at the genome level in more than 30 angiosperms (Bai et al., 2002; Meyers et al., 2003; Monosi et al., 2004; Zhou et al., 2004; Yang et al., 2006, 2008a,b; Ameline-Torregrosa et al., 2008; Mun et al., 2009; Porter et al., 2009; Chen et al., 2010; Li J. et al., 2010; Li X. et al., 2010; Guo et al., 2011; Jupe et al., 2012; Lozano et al., 2012; Luo et al., 2012; Tan and Wu, 2012; Jupe et al., 2013; Lin et al., 2013; Wan et al., 2013; Andolfo et al., 2014; Arya et al., 2014; Kim et al., 2014; Shao et al., 2014; Wu et al., 2014;Yu et al., 2014; Wei C. et al., 2016; Qian et al., 2017; Xiang et al., 2017). Comparative studies of the evolutionary history of NBS-encoding genes were further performed in a number of clades over recent years to illuminate the evolutionary characteristics of NBS-encoding genes. For instance, frequent gene losses and a limited number of gene duplications were pointed out in studies targeting *Cucumis sativus, C. melo*, and *Citrullus lanatus* of the Cucurbitaceae family (Lin et al., 2013). A comparative genomic analysis of four Poaceae species revealed that the number of NBS-encoding genes in *Zea mays* was only half that in *Sorghum bicolor* and *Brachypodium distachyon* and a fourth of that in *Oryza sativa* (Li et al., 2016). Gene loss and retention patterns and a pattern of gene expansion followed by contraction were identified in various species in the Brassicaceae family (Peele et al., 2014; Zhang et al., 2016). In the Fabaceae and Rosaceae, gene expansions were also the most frequently observed evolutionary pattern (Shao et al., 2014; Jia et al., 2015; Zhang et al., 2016).

Oilseed rape (*Brassica napus*, A_n_A_n_C_n_C_n_, 2n = 38) is an allopolyploid that originated from spontaneous hybridization events between the two diploid *Brassica* species *B. rapa* (A_r_A_r_, 2n = 20) and *B. oleracea* (C_o_C_o_, 2n = 18) in the last 10,000 years (Nagaharu, 1935; Chalhoub et al., 2014). Despite the short domestication history of rapeseed (∼300–400 years; Gómez-Campo and Prakash, 1999), it is today one of the most important oil crops worldwide. Hybridization and polyploidisation often produce species with superior resistances or environmental tolerances relative to their progenitor species (Leitch and Leitch, 2008). The exact mechanisms responsible for this effect are unknown, but in rapeseed could result from hybridization, polyploidisation or domestication processes shaping genome evolution. The evolutionary impact of these processes on NBS-encoding genes, which are major players in plant disease resistance, is hence of particular interest. With the availability of genomic data for *B. napus* (Chalhoub et al., 2014) and its two progenitor species *B. rapa* (Wang et al., 2011) and *B. oleracea* (Liu et al., 2014; Parkin et al., 2014), NBS-encoding genes can be systematically investigated to elucidate their role in contributing to differences in disease resistance between the three species, and help to decipher the mechanisms underlying disease resistance in *B. napus*. Previous studies have identified and compared numbers, types and locations of NBS-encoding genes in *B. rapa, B. oleracea*, and *B. napus*. Chalhoub et al. (2014) found 425 NBS-LRRs in *B. napus* (245 in the C genome and 180 in the A genome), similar numbers were reported for the *B. rapa* and *B. oleracea* genomes but with only 75% conservation of synteny. Alamery et al. (2017) identified 641 NBS-LRRs in *B. napus* in total, of which only 365 were intact and hence putatively functional.Yu et al. (2014) identified 239 NBS-LRRs in *B. oleracea*, which was updated to 556 NBS-LRRs in the *B. oleracea* pan-genome (Bayer et al., 2018).

In the present work, a genome-wide characterization of NBS-encoding genes was performed in *B. rapa, B. oleracea*, and *B. napus*. Multiple approaches were utilized to assess the genome architecture and evolutionary characteristics of NBS genes, including genomic distribution, homologous genes, sequence similarities, selection signals, phylogenetic relationships and expression patterns. Resistance genes were also co-localized with previously identified quantitative trait loci (QTL) conferring resistance against major oilseed rape pathogens blackleg (*Leptosphaeria maculans*), clubroot (*Plasmodiophora brassicae*) and *Sclerotinia* stem rot (*Sclerotinia sclerotiorum*), highlighting potential disease resistance candidate genes for future work. This analysis provided genome level insights into the evolution of disease resistance genes in *B. napus*, shedding light on possible mechanisms of disease resistance for future disease resistance breeding in rapeseed.

## Materials and Methods

### Identification of NBS-Encoding Genes in *B. napus, B. rapa*, and *B. oleracea*

The entire genome sequences and annotation data for *B. rapa* (Wang et al., 2011), *B. oleracea* (Liu et al., 2014; Parkin et al., 2014) and *B. napus* (Chalhoub et al., 2014) were downloaded from the BRAD database^1^. NBS-encoding genes in the three species were identified by using the amino acid sequence of the Pfam NB-ARC domain (PF00931) as a “blastp” query against all known protein sequences, via the HMMER V3.0 program with “trusted cutoff” as the threshold (Finn et al., 2011). The obtained hits were further submitted to the Pfam website^2^ to verify the presence of the NB-ARC domain. Those proteins with verified NB-ARC domains were further subdivided into groups based on the structure of the N-terminal and C-terminal domains of the protein. By using the Pfam database^3^, SMART protein motif analyses^4^, and the COILS program^5^, the presence or absence of TIR, CC, LRR domains of NBS-encoding genes was identified, which was used to classify the NBS-encoding genes into different groups.

### Location Anchoring and Gene Cluster Analysis of NBS-Encoding Genes in *B. rapa, B. oleracea*, and *B. napus*

The chromosome size and physical position of NBS-encoding genes were downloaded from the BRAD database^6^ for location anchoring of NBS-encoding genes. The visualization of NBS-encoding genes on 10, 9, and 19 chromosomes (assembled pseudomolecules) of *B. rapa, B. oleracea*, and *B. napus* was drawn by “PhenoGram Plot”.^7^ According to the gene cluster definition proposed by Richly et al. (2002) and Meyers et al. (2003), two or more genes located within eight ORFs of each other were treated as a gene cluster.

### Detection of Duplicated Genes Within Each Species and Identification of Homologous Gene Pairs Between *B. rapa, B. oleracea*, and *B. napus*

To detect the duplicated NBS-encoding genes within individual species and to identify homologous gene pairs between species, the BLASTP program of Blast2GO (Conesa et al., 2005) was employed using protein sequences with stringent parameters, i.e., *E*-value cutoff (*e* = 0.0) (Ariyarathna and Francki, 2016). The gene pairs across genomes were visualized using the Circos software (Krzywinski et al., 2009). If no homologous gene was found for a particular gene using this stringent criterion, the top hit was selected and designated as the most similar gene.

### Multiple Alignments and Phylogenetic Analysis of NBS-Encoding Genes

Multiple alignments of amino acid sequences were performed using the program “Muscle” with default options (Thompson et al., 1994). The software RAxML was then used to construct phylogenetic trees based on the Maximum Likelihood (ML) method (Stamatakis, 2015). Phylogenetic trees were drawn by the software Figtree^8^.

### Non-synonymous/Synonymous Substitution (Ka/Ks) Ratios of Homologous Gene Pairs

Protein sequence variation between each homologous pair was firstly calculated via sequence alignment using the program Muscle with default options (Thompson et al., 1994). Based on the alignment results, the number of nucleotide variants was then calculated by reverse translation of the protein sequence to nucleotides. The ratio of nucleotide variants was calculated by the formula: ratio of nucleotide variants = number of variant nucleotides/the number of aligned nucleotides (gaps were not considered). To investigate selective pressure on NBS-encoding genes, the ratio of non-synonymous substitutions to synonymous substitutions (*Ka/Ks*) was calculated. Firstly, the protein sequences of NBS-encoding genes in each homologous gene family were aligned using the program Muscle with default options (Thompson et al., 1994). Then, non-synonymous substitutions (*Ka*) and synonymous substitutions (*Ks*), and the ratio between them (*Ka/Ks*) were calculated in each homologous gene family using the tool yn00 in the paml package (Yang, 1997). Positive and negative selective pressure were justified as a *Ka/Ks* ratio > 1 and *Ka/Ks* ratio < 1, respectively, while a ratio of 1 indicated neutral evolution (Yang, 2007). *Ka/Ks* ratio cutoffs of > 1.2 and < 0.8 were chosen to identify positively and negatively selected genes, respectively, with slight modification of the method of Mayer et al. (2011).

### RNA-seq Data Analysis of NBS-Encoding Genes

In order to analyze the expression of the NBS-encoding genes, RNA-seq data of NBS-encoding genes of *B. rapa, B. oleracea* and *B. napus* that was generated earlier and submitted to the GEO database was downloaded. RNA-seq data of five tissues (stem transcripts, root transcripts, leaf transcripts, flower transcripts and seed transcripts) of *B. napus, B. rapa*, and *B. oleracea* were obtained from Tong et al. (2013); Liu et al. (2014), and Miao et al. (2016), respectively. Fragments per kilobase of exon model per million mapped reads (FPKM) were calculated for NBS-encoding genes. The FPKM values were log2 transformed, and a hierarchical cluster was created using the Genesis software using the hierarchical clustering algorithm (Eisen et al., 1998).

### Co-localization of NBS-Encoding Genes and Mapped Quantitative Resistance Loci Against Three Major Diseases of *B. napus*

Resistance quantitative trait loci for blackleg (*Leptosphaeria maculans* and *L. biglobosa*), clubroot (*Plasmodiophora brassicae*), and *Sclerotinia* stem rot (*Sclerotinia sclerotiorum*), were collected from the literature (Hirai et al., 2004; Suwabe et al., 2006; Zhao et al., 2006; Delourme et al., 2008; Sakamoto et al., 2008; Kaur et al., 2009; Nagaoka et al., 2010; Raman et al., 2012; Jian et al., 2013; Kato et al., 2013; Mei et al., 2013; Wei et al., 2014; Wei L. et al., 2016; Larkan et al., 2016; Li et al., 2016; Sanjaya et al., 2016; Wu et al., 2016; Kumar et al., 2018; Pang et al., 2018) (Supplementary Table S1). The QTL intervals were aligned to the reference genomes of *B. napus*^9^ by BLAST analysis of the sequences of SSR primers or probe sequences linked to the QTLs, using the method of Cai et al. (2012) with slight modifications. The alignment criteria were set to allow three mismatches and one gap for a given primer pair. When a marker had multiple amplification loci on the same chromosome, an accurate position for a particular locus was determined manually by referring to the physical positions of its upstream and downstream markers.

## Results

### Identification of NBS-Encoding Genes in *B. napus, B. rapa*, and *B. oleracea*

**FIGURE 1:**
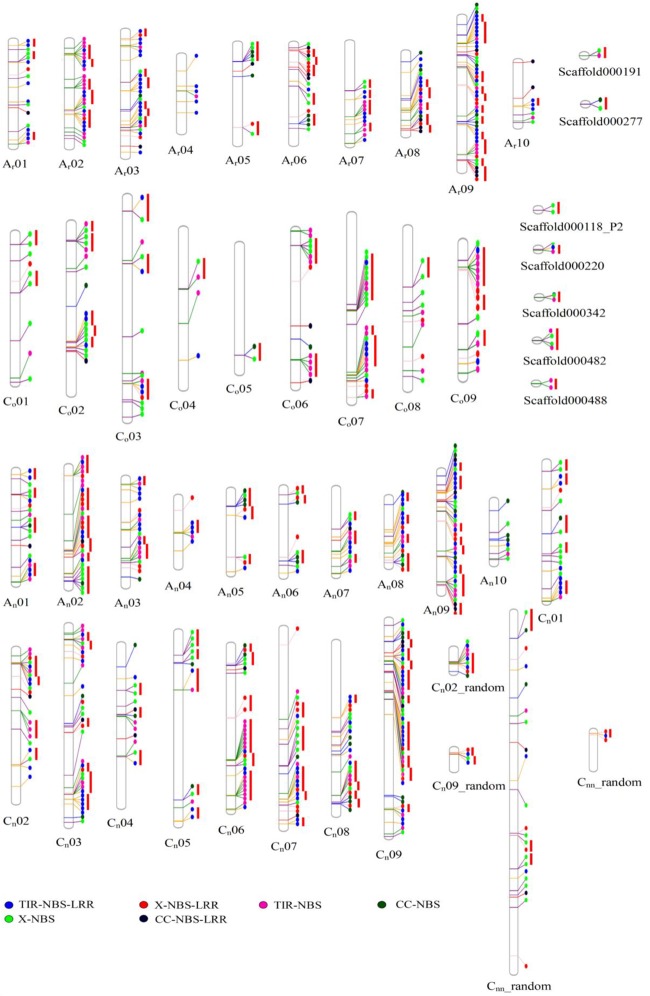
The physical location of NBS-encoding genes in A_r_ genome of *B. rapa*, C_o_ genome of *B. oleracea* and A_n_- and C_n_- subgenomes of *B. napus*. The circles in different colors represented types of NBS-encoding genes.

A total of 464, 202, and 146 NBS-encoding genes were identified in *B. napus, B. rapa*, and *B. oleracea*, respectively (Table 1 and Figure 1). The genome of *B. napus* contained 116 additional NBS-encoding genes when compared to the total number of NBS-encoding genes present in the two progenitor species, a considerable increase (33.33% more than the total number of NBS-encoding genes in the two progenitor species). According to gene structure and protein motifs, we categorized these NBS-encoding genes into six different classes: TNL (134, 76, and 19 for *B. napus, B. rapa*, and *B. oleracea*, respectively), CNL (16, 3, and 19), XNL (18, 13, and 61), TN (37, 45, and 85), CN (18, 3, and 45) and XN (37, 63, and 120) (Table 1). Except for the CNL group, which showed the same gene number in *B. napus* compared to the total in the two progenitor species, all the other five classes of NBS-encoding genes contained more genes in *B. napus* than in the two progenitor species. The greatest increase in *B. napus* relative to the progenitors was for the TNL gene class (39 additional genes).

**Table 1:** The number of NBS-encoding genes in the genomes of *B. rapa, B. oleracea*, and *B. napus.*

Predicted protein domains	*B. rapa*	*B. oleracea*	*B. napus*
NBS-encoding genes	202	146	464
NBS-LRR type	110	35	214
TIR-NBS-LRR	76	19	134
non-TIR-NBS-LRR	34	16	80
CC-NBS-LRR	16	3	19
X-NBS -LRR	18	13	61
NBS-only	92	111	250
TIR-NBS	37	45	85
Non-TIR-NBS	55	66	165
CC-NBS	18	3	45
X-NBS	37	63	120
Average length of NBS-encoding genes (Minimum∼Maximum) (nt)	4198 bp (518∼15805)	3553 bp (164∼13901)	3982 bp (83∼24393)
Average exon number of NBS-encoding genes (Minimum Maximum)	4.52 (1 22)	4.25 (1 32)	5.31 (1 40)


### Sequence Comparison of NBS-Encoding Genes Between *B. napus* and Its Progenitor Species

The size range of NBS-encoding genes in *B. napus* was much greater than NBS-encoding genes in the progenitors (83–244393 nt; as compared to 518–15805 nt and 164–13901 nt in *B. rapa* and *B. oleracea*, respectively, Table 1). *B. napus* also had more exons on average than the progenitor species: on average 5.31 exons compared to 4.52 and 4.25 in *B. rapa* and *B. oleracea* respectively, and a maximum of 40 exons compared to 22 and 32 in *B. rapa* and *B. oleracea* respectively.

In order to compare variation in NBS-encoding genes between *B. napus* and its progenitors, BLAST was used for the 464 NBS-encoding genes in *B. napus* against the NBS-encoding genes in the two progenitor species, and the best matching gene pairs were compared for total gene length and number of exons. On average, a 219 bp decrease in sequence length was observed for NBS-encoding genes in *B. napus* when compared with the corresponding best match for NBS-encoding genes in the progenitor species (a total of 10,1719 bp decrease). By contrast, the average number of exons in *B. napus* relative to its progenitor species increased by 0.91 (an additional 421 exons genome-wide). These results support a trend of overall decrease in sequence length but an increase in number of exons for NBS-encoding genes in *B. napus* after its formation from the progenitor species.

A total of 1456 fragments (>10 bp) from 412 NBS-encoding genes in *B. napus* did not align to the corresponding best matching NBS-encoding genes in the progenitor species. These fragments were subsequently aligned to the whole genome sequences of *B. rapa* and *B. oleracea* using BLAST. Of these, 1083 fragments (74%) from 372 NBS-encoding genes of *B. napus* could be completely or partially aligned with the genomic sequences of the two progenitors (*e* = 1.0). These results suggest abundant non-homologous recombination between the NBS-encoding genes and other genomic sequences from progenitor species during the processes of hybridization and polyploidization, or the subsequent domestication. The remaining 373 fragments not only did not align to the progenitor genomes, but also seldom hit with other species on NCBI (*e* = 10). However, 8 of the 373 fragments matched known TE motifs from *A. thaliana*, suggesting that some of these fragments may have formed by TE-mediated mutation processes (Wicker et al., 2016). Additionally, 376 fragments with no best hits and low homology (<35%) with the progenitor genomes were further extracted for alignments using BLAST (*e* = 10) against all species on NCBI. 114 sequences showed no hit with any species, but three fragments matched with known TE motifs from *A. thaliana*, suggesting these sequences may also have arisen via mutational processes and TE insertion within *B. napus*. Of the 262 sequences which could be anchored, 92 sequences matched best with the genomic sequences of the progenitors (after use of a less stringent *e* value), suggesting drastic variation within these sequences, and 84 sequences had a highest match with the genomes of other Brassicaceae species (46.3% from *Raphanus sativus* and *Arabis alpine*). A further 31 sequences had top matches outside the Brassicaceae family: 23 sequences were best matched with some microorganisms; 26 sequences were best matched with some worms, e.g., nematodes and trematodes; and 6 sequences were best matched with some other animals.

### Genomic Distribution of NBS-Encoding Genes Along Chromosomes in the Three Species

**FIGURE 2:**
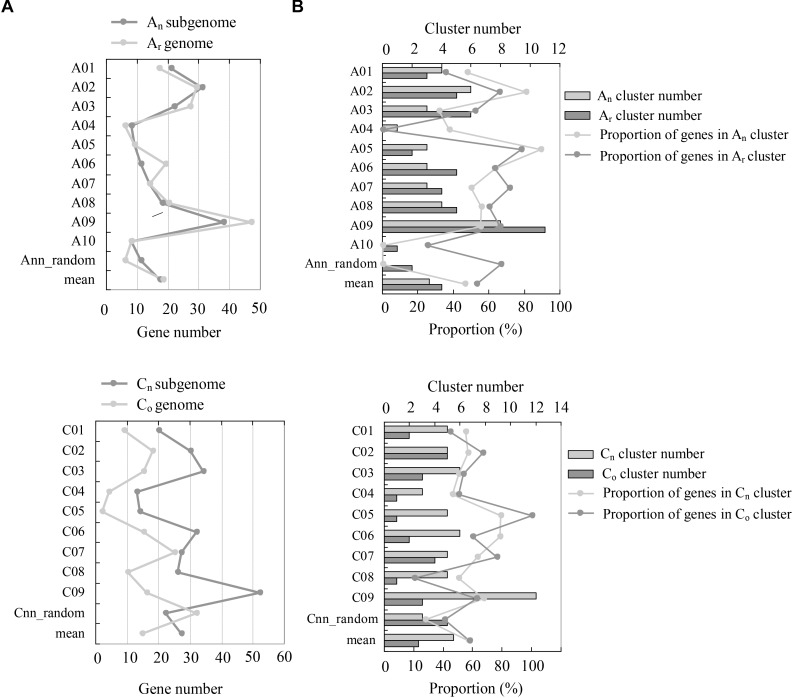
Comparison of NBS-encoding genes between the subgenomes of *B. napus* (A_n_A_n_C_n_C_n_) and the genomes of *B. rapa* (A_r_A_r_) and *B. oleracea* (C_o_C_o_). **(A)** The comparison of gene numbers between the genomes of A_r_ and A_n_, and C_o_ and C_n_. The *y*-axis indicates the number of NBS-encoding genes located on each individual chromosome, and the *x*-axis represents the chromosomes. **(B)** The comparison of NBS-encoding gene clusters between the A_r_ and A_n_, and C_o_ and C_n_ genomes. The *y*-axis represents the chromosomes, while the *x*-axis represents the number of gene clusters and the proportion of genes located in each gene cluster, respectively.

The genomic distribution of NBS-encoding genes between recent allopolyploid *B. napus* (A_n_A_n_C_n_C_n_) and its progenitors *B. rapa* (A_r_A_r_) and *B. oleracea* (C_o_C_o_) was determined (Figure 2). Hundred and ninety one NBS-encoding genes were identified in the A_n_-subgenome of *B. napus*, which was quite similar to the number of NBS-encoding genes (202) in the A_r_ genome of *B. rapa*. The distribution of these genes on different chromosomes in the A genome was uneven, although the A_n_-subgenome of *B. napus* and the A_r_ genome of *B. rapa* showed similar genomic distributions. Chromosome A09 contained the most NBS-encoding genes in both A genomes, with 38 (19.9%) and 47 (23.27%) NBS-encoding genes located on A_n_09 and A_r_09, respectively. 273 NBS-encoding genes were identified in the C_n_-subgenome of *B. napus*, which was a considerably greater number than were identified in the C_o_ genome of *B. oleracea* (146). NBS-encoding genes were also unevenly distributed between the C_n_-subgenome and C_o_ genomes. The chromosome with the most NBS-encoding genes differed between the two C genomes: C_n_9 and C_o_7 contained the highest numbers of NBS-encoding genes in each genome, with 52 (19.26%) and 25 (17.12%) NBS-encoding genes, respectively.

Resistance genes residing in clusters may facilitate the evolutionary process of novel resistance gene production via increased tandem duplication and gene recombination. Using the definition of a gene “cluster” defined by Richly et al. (2002) and Meyers et al. (2003) as two or more genes falling within eight ORFs, we identified 44, 27, and 90 NBS-encoding gene clusters in *B. rapa, B. oleracea*, and *B. napus*, which included 117, 81, and 256 NBS-encoding genes respectively in the three species (57.92% in *B. rapa*, 55.48% in *B. oleracea* and 55.53% in *B. napus*). Both the number of gene clusters and the number of genes within clusters in *B. napus* were much more than the sum of the two progenitor species. Chromosomes A_n_09 and C_n_9 in *B. napus* possessed the most abundant gene clusters genome-wide. In the two progenitor species, chromosomes A_r_09 and C_o_7 possessed the most abundant gene clusters in *B. rapa* and *B. oleracea*, respectively.

### Comparative Homology of NBS-Encoding Genes Between Genomes

**FIGURE 3:**
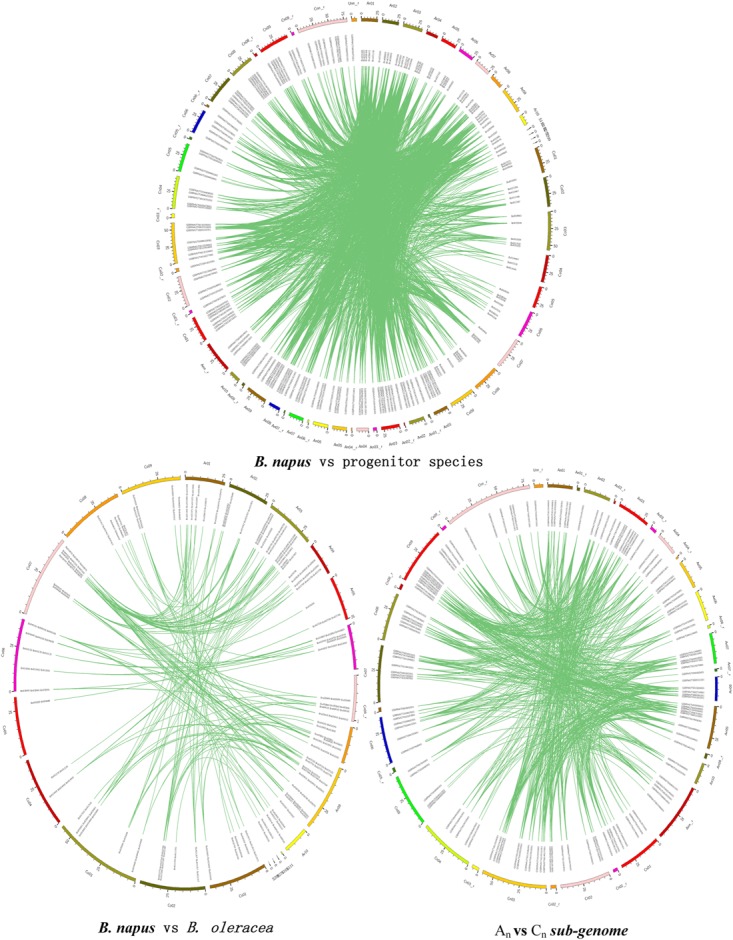
NBS-encoding gene homologs between the *B. napus, B. Rapa*, and *B. oleracea* genomes. Colored bars represent chromosomes of the three species. Green lines indicate the relationships of homologous gene pairs between genomes.

NBS-encoding genes were assessed for homology between the three species’ genomes (Table 2). Three hundred and forty six NBS-encoding genes of *B. napus*, forming 3454 homologous gene pairs, were identified between *B. napus* and its two progenitor species, with uneven distribution between chromosomes (Figure 3). Chromosome C_o_9 had the highest number of homologous gene pairs (432 pairs) and C_o_4 possessed the fewest homologous gene pairs (52 pairs). Of these homologous gene pairs, 70.9 and 50.2% of NBS-encoding genes in *B. napus* were homologous with those from *B. rapa* and *B. oleracea*, respectively (Table 2). For the 202 NBS-encoding genes in *B. rapa*, 176 (87.1%) were homologous with NBS-encoding genes in *B. napus*, while only 97 (66.4%) of NBS-encoding genes in *B. oleracea* were homologous with NBS-encoding genes in *B. napus* (Table 2). Although the remaining NBS-encoding genes of *B. rapa* and *B. oleracea* lacked clear homologous NBS-encoding gene partners in *B. napus*, 24 (11.9%) and 45 (30.8%) of genes still showed best hits to NBS-genes in *B. napus*, and only 2 (1.0%) and 4 (2.7%) of NBS-genes in *B. rapa* and *B. oleracea* had altered NBS-domains in *B. napus* (Table 2).

**Table 2:** Homology analysis of NBS-encoding genes between *B. napus* (2n = A_n_A_n_C_n_C_n_), *B. rapa* (2n = A_r_A_r_) and *B. oleracea* (2n = C_o_C_o_).

Species/genomes	Total no. NBS-encoding genes	Target species/genomes	Homologous genes found	No homologues but top hit was an NBS-encoding gene	No homologues and top hit was not an NBS-encoding gene
A_n_A_n_C_n_C_n_	464	A_r_A_r_	329 (70.9%)	112 (24.1%)	23 (5.0%)
A_n_A_n_C_n_C_n_	464	C_o_C_o_	233 (50.2%)	110 (23.7%)	121 (26.1%)
A_r_A_r_	202	A_n_A_n_C_n_C_n_	176 (87.1%)	24 (11.9%)	2 (1.0%)
A_r_A_r_	202	C_o_C_o_	128 (63.4%)	57 (28.2%)	17 (8.4%)
C_o_C_o_	146	A_n_A_n_C_n_C_n_	97 (66.4%)	45 (30.8%)	4 (2.7)
C_o_C_o_	146	A_r_A_r_	79 (54.1%)	63 (43.2%)	4 (2.7%)
A_n_	191	C_n_	129 (67.5%)	54 (27.3%)	8 (4.2%)
C_n_	270	A_n_	149 (54.8%)	116 (43.0%)	5 (1.9%)


*The parameter *e* = 0.0 was employed to screen for homologous gene pair. If no homologous gene was found for a particular gene using this stringent criterion, the top hit was selected and designated as the most similar gene.*

A total of 811 homologs of NBS-encoding genes were identified between the two progenitor species (Figure 3). Of these, 128 NBS-encoding genes (63.4%) in *B. rapa* were homologous to NBS-encoding genes in *B. oleracea*, and 79 NBS-encoding genes (54.1%) in *B. oleracea* were homologous to NBS-encoding genes in *B. rapa*. As expected, NBS-encoding gene sequences varied more between the two progenitor species than between the progenitors and *B. napus*, due to the longer evolutionary divergence time between the two progenitor species.

Overall, 1509 NBS-encoding homologous gene pairs were identified between the A_n_- and C_n_-subgenomes of *B. napus* (Figure 3), which was almost twice the number observed between the A_r_ and C_o_ genomes of *B. rapa* and *B. oleracea*. This result indicated the genetic assimilation of NBS-encoding genes might occur between A_n_ and C_n_ sub-genomes during the formation and domestication of *B. napus*. The proportions of genes with homologs in the A_n_ subgenome (67.5%, 129 genes) and C_n_ subgenome (54.8%; 149 genes) of *B. napus* were similar to the proportions of genes with homologs between the two progenitor species.

### Analysis of Selective Pressure on NBS-Encoding Genes

Using NBS-encoding gene homologs identified between genomes, we calculated *Ks* (synonymous substitution rate) and *Ka* (non-synonymous substitution rate) values for homologous gene pairs. In total, 3454 homologous gene pairs were identified between *B. napus* and two progenitor species, including 2774 homologous gene pairs between *B. napus* and *B. rapa* and 680 homologous gene pairs between *B. napus* and *B. oleracea*. Overall, 98.5 and 97.34% of the homologous gene pairs between *B. napus* and the two progenitor species showed *Ka/Ks* ratios less than 1 (99.4 and 94.9% for *B. rapa* and *B. oleracea*, respectively) and 0.8 (98.27 and 93.53% for *B. rapa* and *B. oleracea*, respectively) (Table 3), and the majority of homologous genes showed *Ka/Ks* ratios ranging from 0.3 to 0.4. These results indicate the presence of purifying selection (selection against change) on most of the NBS-encoding genes following the divergence of *B. napus* from the progenitor species. The mean *Ka/Ks* value for all homologous gene pairs between *B. napus* and *B. rapa* was 0.38, slightly but not significantly lower than the *Ka/Ks* value of 0.40 between *B. napus* and *B. oleracea*. A total of 35 homologous gene pairs between *B. napus* and its two progenitor species showed *Ka/Ks* ratios of more than 1, of which 15 homologous gene pairs showed *Ka/Ks* ratios of more than 1.2, suggesting positive selection for beneficial mutations. Gene ontology (GO) analysis of these genes revealed that the majority of these genes had molecular functionality in ADP binding properties and were involved in signal transduction and defense response processes.

**Table 3:** *Ka/Ks* ratio of homologous gene pairs for NBS-encoding genes between the genomes of *B. napus* and its progenitor species *B. rapa* and *B. Oleracea.*

Homologous gene pairs	No. gene pairs	mean ± SD	Min∼max	Negative selection			Positive selection	
					
				<0.25	<0.8	<1	>1	>1.2
*B. napus* vs. progenitor species	3454	0.39 ± 0.20	0.03∼6.75	394 (11.41%)	3362 (97.34%)	3402 (98.50%)	35 (1.01%)	15 (0.43%)
*B. napus* vs. *B. oleracea*	680	0.40 ± 0.15	0.03∼2.20	85 (12.5%)	636 (93.53%)	645 (94.85%)	20 (2.94%)	8 (1.18%)
*B. napus* vs. *B. rapa*	2774	0.38 ± 0.16	0.06∼6.75	309 (11.14%)	2726 (98.27%)	2757 (99.39%)	15 (0.54%)	7 (0.25%)


### Analyses of Phylogenetic Patterns of NBS-Encoding Genes Between *B. napus* and Its Progenitor Species

Whole genome duplication is thought to be a source of complexity and diversity for plant species that may allow them to adapt to changing environmental conditions. To investigate the phylogenetic relationships of NBS-encoding genes among genomes, the coding sequences of all NBS-encoding genes in *B. napus, B. rapa*, and *B. oleracea* were analyzed and compared, and a phylogenetic tree was constructed (Supplementary Figure S1). In the phylogenetic tree, at least six evolutionary patterns were observed.

The first evolutionary pattern observed was that of straightforward inheritance of genes from progenitor species *B. rapa* and *B. oleracea* to their allopolyploid progeny species *B. napus*. This evolutionary pattern was commonly observed for NBS-encoding genes in *B. napus*. For instance in one cluster (shown in Supplementary Figure S2a), seven NBS-encoding genes of *B. napus* individually grouped with eight homologous NBS-encoding genes from both *B. rapa* and *B. oleracea*. In the second evolutionary pattern, NBS-encoding genes in *B. napus* originated from a single progenitor species (Supplementary Figure S2b). In one example (Supplementary Figure S2b), 11 NBS-encoding genes in *B. napus* grouped with 13 homologous NBS-encoding genes from *B. rapa*. The third evolutionary pattern found was a large scale expansion of NBS-encoding genes in *B. napus* relative to the progenitor species. A typical gene cluster with this pattern is shown in Supplementary Figure S2c, where 13 NBS-encoding genes of *B. napus* were grouped with only one NBS-encoding gene from *B. rapa*. The fourth evolutionary pattern was the loss of NBS-encoding R-genes from progenitors in *B. napus*. For instance in one cluster shown in Supplementary Figure S2d, nine NBS-encoding genes from the progenitor species were grouped with only three NBS-encoding R-genes from *B. napus*. The fifth evolutionary pattern observed is that of different sequence divergence rates between NBS-encoding genes. Some gene clusters in the phylogenetic tree had longer branch lengths than others, which suggested different sequence divergence rates between gene clusters. For instance, in a cluster shown in Supplementary Figure S2e, one NBS-encoding gene of *B. napus* showed an extremely long branch length relative to the other genes in the cluster, suggesting a more rapid sequence divergence rate for this gene. A sixth (rare) evolutionary pattern of gene loss from the progenitor species to *B. napus* was also observed for two NBS-encoding genes (Supplementary Figure S1). As individual gene clusters commonly exhibited more than one evolutionary pattern, relative prevalence of each type was difficult to assess. However, we observed at least six patterns for the evolution of NBS-encoding genes in *B. napus*, suggesting a complicated evolutionary trajectory for NBS-encoding genes despite the relatively short history of this young allopolyploid species.

### Comparative Expression of NBS-Encoding Genes in *B. rapa, B. oleracea*, and *B. napus*

As well as variation in gene copy number and in gene sequences, variation in gene expression is also an important aspect of gene evolution. Relative expression data for the NBS-encoding genes was collected from the root, stem, leaf, flower and seed tissues of *B. rapa, B. oleracea*, and *B. napus* (Supplementary Table S1). According to the gene expression level, 28 gene expression models were determined for the NBS-encoding genes in the three species (Table 4). The first five gene expression models encompassed 62.5% of NBS-encoding genes in *B. napus* (290 genes). The most common model was for genes to be expressed more highly in the root only, followed by higher gene expression in root and leaf, then higher expression in leaf tissue only, then higher expression in root and flower, then higher expression in stem and leaf. In *B. rapa*, these first five expression models covered 121 NBS-encoding genes (59.9%). The proportion of genes fitting each of the five expression models in *B. rapa* were sorted from larger to smaller as follows: higher expression in root only, higher expression in stem and leaf, higher expression in root and stem, higher expression in root, stem and leaf, and higher expression in root and seed. In *B. oleracea*, the first five expression models covered 105 NBS-encoding genes (71.9%). The proportion of genes fitting each of the five expression models were sorted from larger to smaller as follows: higher expression in root only, no higher expression in any of the five tissues, higher expression in root and leaf, higher expression in root and stem, and higher expression in root and flower.

**Table 4:** Expression patterns of NBS-encoding genes in *B. napus* (2n = AACC), *B. rapa* (2n = AA), and *B. oleracea* (2n = CC) root, stem, leaf, flower, and seed tissues.

Patterns	Type^a^					*B. napus*		*B. rapa*		*B. oleracea*	
				
	Root	Stem	Leaf	Flower	Seed	gene number	%	gene number	%	gene number	%
1	+	-	-	-	-	112	24.1	47	23.3	48	32.9
2	+	-	+	-	-	52	11.2	5	2.5	13	8.9
3	-	-	+	-	-	47	10.1	8	4.0	5	3.4
4	+	-	-	+	-	46	9.9	8	4.0	9	6.2
5	-	+	+	-	-	33	7.1	24	11.9	4	2.7
6	-	-	-	+	-	25	5.4	3	1.5	0	0
7	-	-	+	+	-	22	4.7	9	4.5	0	0
8	+	+	-	-	-	18	3.9	21	10.4	12	8.2
9	+	-	+	+	-	18	3.9	1	0.5	0	0
10	-	-	-	-	-	17	3.7	8	4.0	23	15.8
11	-	+	-	-	-	11	2.4	2	1.0	7	4.8
12	+	+	+	-	-	11	2.4	16	7.9	0	0
13	-	+	-	+	-	8	1.7	4	2.0	1	0.7
14	-	+	+	+	-	8	1.7	8	4.0	0	0
15	+	-	-	-	+	7	1.5	13	6.4	2	1.4
16	+	-	+	-	+	5	1.1	0	0	1	0.7
17	-	+	-	-	+	4	0.9	3	1.5	3	2.1
18	-	-	+	-	+	4	0.9	2	1.0	4	2.7
19	+	+	-	+	-	4	0.9	7	3.5	0	0
20	-	-	-	-	+	3	0.7	2	1.0	4	2.7
21	+	-	-	+	+	2	0.4	2	1.0	1	0.7
22	-	+	+	-	+	2	0.4	3	1.5	2	1.4
23	-	-	-	+	+	1	0.2	4	2.0	3	2.1
24	+	+	-	-	+	1	0.2	1	0.5	1	0.7
25	-	+	-	+	+	1	0.2	1	0.5	1	0.7
26	-	-	+	+	+	1	0.2	0	0	1	0.7
27	+	+	+	+	-	1	0.2	0	0	0	0
28	-	+	+	+	+	0	0	0	0	1	0.7


*^*a*^Expression level was normalized to a ratio ranging from -3.0 to 3.0. Positive signs represent ratios greater than 0.0 (higher expression) and negative signs represent ratios less than 0.0 (lower expression).*

**FIGURE 4:**
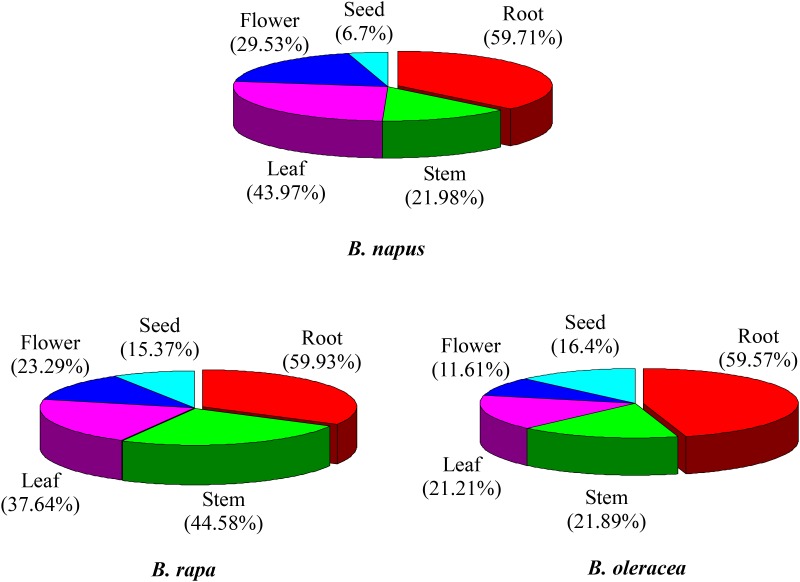
Expression analysis of NBS-encoding genes in tissues of *B. rapa, B. Oleracea*, and *B. napus*. The proportions represent higher expression of NBS-encoding genes in each tissue.

The majority of the NBS-encoding genes showed higher expression levels in the root in all of the three species, accounting for 59.9, 59.6, and 59.7% of the NBS-encoding genes in *B. rapa, B. oleracea*, and *B. napus* (Figure 4). In contrast, the proportion of NBS-encoding genes with higher expression in the seed was low in all the three species, accounting for 15.4, 16.4, and 6.7% of the NBS-encoding genes in *B. rapa, B. oleracea*, and *B. napus* (Figure 4). These results revealed that the NBS-encoding genes were more likely to be highly expressed in root tissue, which reflects that the roots might show stronger stress responses (or be under greater disease stress) relative to the other tissues.

### Comparative Analysis of the Identified NBS-Encoding Genes and Mapped Quantitative Resistance Loci of Three Major Diseases of *B. napus*

Blackleg (*Leptosphaeria maculans* and *L. biglobosa*), clubroot (*Plasmodiophora brassicae*), and *Sclerotinia* stem rot (*Sclerotinia sclerotiorum*) are three key pathogens affecting *B. napus*. Here, we collected mapped resistance QTLs against these three major disease-causing pathogens, to identify colocalization of the identified NBS-encoding genes and these QTLs. A total of 170 chromosome intervals distributed on the 19 chromosomes of *B. napus*, were obtained by BLAST analysis of the flanking markers of known QTLs against the reference genome of *B. napus*, and included 81 loci associated with blackleg resistance, 19 loci associated with clubroot resistance, and 70 loci associated with *Sclerotinia* stem rot resistance (Supplementary Table S2). Of these QTL mapped to chromosome intervals, 71 were co-located with NBS-encoding genes identified in the present study (204 genes; Supplementary Table S3). Each of these 71 intervals contained from 1 to 18 NBS-encoding genes (Supplementary Table S3).

Of the 204 NBS-encoding genes located within the mapped QTLs, 165 NBS-encoding genes could be mapped within QTLs for blackleg resistance, 51 NBS-encoding genes could be mapped within the QTLs for clubroot resistance, and 41 NBS-encoding genes could be mapped within the QTLs for *Sclerotinia* stem rot resistance (Supplementary Tables S3, S4). Hundred and fifty four genes were co-located with resistance QTLs against a single disease. Of which, 139 genes were co-located with a resistance QTL identified for a single disease, including 105, 18, and 16 genes co-located with resistance QTLs against blackleg, clubroot and *Sclerotinia* stem rot, respectively (Supplementary Table S4), and 15 genes were co-located with at least two overlapping QTLs for the same disease from different studies, including 13 genes within overlapping resistance QTLs against blackleg, and two genes within overlapping resistance QTLs against clubroot (Supplementary Table S4). A further 47 NBS-encoding genes were co-located with two overlapping resistance QTLs against different diseases, including 25 genes within resistance QTLs against both blackleg and clubroot, 19 genes within resistance QTLs against both blackleg and *Sclerotinia* stem rot, and three genes within resistance QTLs against both clubroot and *Sclerotinia* stem rot (Supplementary Table S4). Three genes were also found within overlapping QTLs against all three diseases (Supplementary Table S4).

## Discussion

### Massive Copy Number Increase in Putative Resistance Genes in *B. napus* Relative to Its Progenitor Species *B. rapa* and *B. oleracea*, Particularly in the C Genome

Despite the fact that *B. napus* is a very recent allopolyploid, <10,000 years old (Chalhoub et al., 2014), we found extremely dynamic evolution of putatively functional resistance genes in this species relative to its progenitor species *B. rapa* and *B. oleracea*. In particular, an additional 116 new NBS-encoding genes were identified (an increase of 33%), the vast majority of which were observed in the *B. napus* C genome relative to the progenitor *B. oleracea* genome. Significant sequence divergence was also observed in 1/3 of the NBS-encoding genes, and clustering patterns were also changed in the *B. napus* C genome relative to in *B. oleracea.* This pattern may be related to the much higher fraction of repetitive sequences and transposable elements in the C genome relative to the A genome (Wang et al., 2011; Chalhoub et al., 2014; Liu et al., 2014; Parkin et al., 2014): resistance genes are often located within more highly repetitive regions and hence undergo more dynamic evolutionary processes as a result, as this facilitates generation of new, potentially useful phenotypic variation for disease resistance. Our results differ significantly from those of Alamery et al. (2017), who found similar numbers of NBS-LRR genes in *B. napus* relative to its progenitors as well as many more genes. However, as we used functional protein coding domains as search queries and ignored pseudogenes, it is likely that the differences between our studies can be explained by this difference in approach. In support of this, 43% of NBS-LRR genes identified by Alamery et al. (2017) were thought to be non-functional in this study. Hence, our results are more likely to reflect functional evolutionary patterns for disease resistance genes in the *B. napus* genome.

#### NBS-Encoding Genes Were Preferentially Expressed in Root Tissues in All Three Species

Roots are an essential organ for a myriad of physiological processes, including water and nutrient uptake, anchoring of plants, storage of assimilates and mechanical support of the above-ground organs. Roots are constantly in contact with the soil microbial community, which consists of mostly beneficial but also pathogenic organisms. Thus, plants have evolved root-specific defense responses against soil-borne pathogens, viruses, pests and oomycetes, including pre-formed physical and chemical barriers and inducible defenses (Dixon, 2001;De Coninck et al., 2015). NBS-encoding genes are the largest class of known disease resistance genes for defense against various pathogens, including bacteria, fungi, oomycetes, viruses, and nematodes. Therefore, it is not surprising that abundant NBS-encoding genes may actually be involved in beneficial root–pathogen interactions in the soil (Okubara and Paulitz, 2005). For instance, the tomato *I-2* gene, a member of the NBS-LRR family, confers resistance to the wilt pathogen *Fusarium oxysporum* f.sp. *lycopersici* (Mes et al., 2000), and is preferentially expressed in lateral root primordia of young roots and in vascular regions of mature roots (Okubara and Paulitz, 2005). Two NBS-LRR genes (*Mi-1* and *Hero A*) from tomato and two NBS-LRR genes (*Gpa2* and *Gro1-4*) from potato individually confer root-specific resistance against root knot nematodes and cyst nematodes (Williamson and Kumar, 2006). Our study found that NBS-encoding genes were predominantly expressed in root tissue in each of *B. rapa, B. oleracea*, and *B. napus*, which is congruent with the greater pressure from micro-organisms on the root than on the other above-ground organs. Future studies may elucidate whether these genes are involved in disease resistance or in beneficial interactions with soil micro-organisms.

### Diverse Patterns Explain the Rapid Evolution of NBS-Encoding Genes in *B. napus* After Formation From Its Diploid Progenitor Species

**FIGURE 5:**
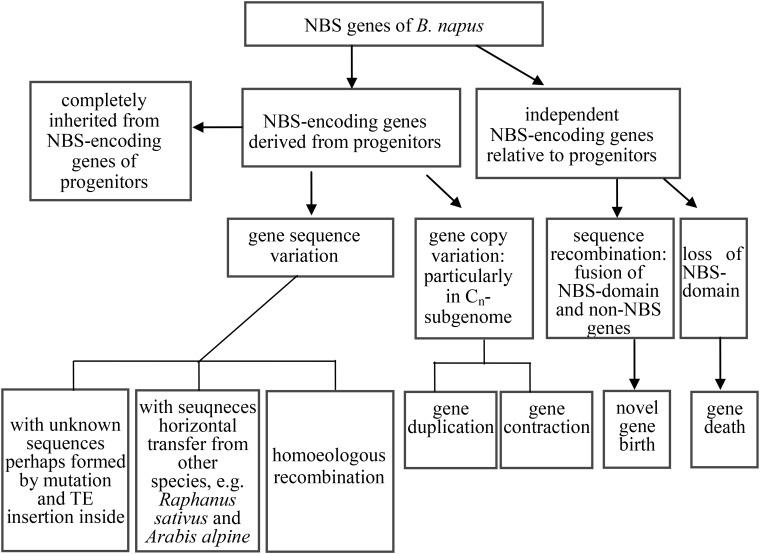
The evolutionary routes of NBS-encoding genes in *B. napus* relative to its two progenitor species *B. rapa* and *B. oleracea*.

Phylogenetic analysis of coding sequences of NBS-encoding genes in the three species revealed at least six distinct evolutionary patterns, falling into three groups. We summarize these evolutionary patterns in Figure 5. The first group represented the straightforward inheritance of NBS-encoding genes directly from the progenitor species to *B. napus* (the first two patterns identified in the results). Within this group, NBS-encoding genes in *B. napus* were inherited directly from the two progenitor species, either as one or more copies from both progenitors or as one or more copies from only one of the two progenitor species. Given that *B. rapa* and *B. oleracea* diverged from each other about 1.44 Mya (Arias et al., 2014), followed by the very recent formation of *B. napus* as an allopolyploid species < 10,000 years ago (Chalhoub et al., 2014), it is not surprising that these two patterns of direct inheritance from both progenitor species as well as inheritance of species-specific genes were both commonly observed.

The second group represented the expansion and contraction of NBS-encoding genes in *B. napus* relative to its two progenitor species (evolutionary patterns 3 and 4 in the Results section). In many species, such as grapevine, poplar, rice, and legumes, large scale expansions and contractions of NBS-encoding genes have also been found (Zhou et al., 2004; Yang et al., 2008a,b; Zheng et al., 2016). Overall, previous research suggests that plant resistance genes have very rapid expansion and contraction rates, as they evolve in response to interactions with pathogens (Faulkner and Robatzek, 2012; Nepal and Benson, 2015). Hence, the expansion and contraction of NBS-encoding genes in *B. napus* is expected to facilitate rapid evolution of resistance, particularly as a response to domestication.

**FIGURE 6:**
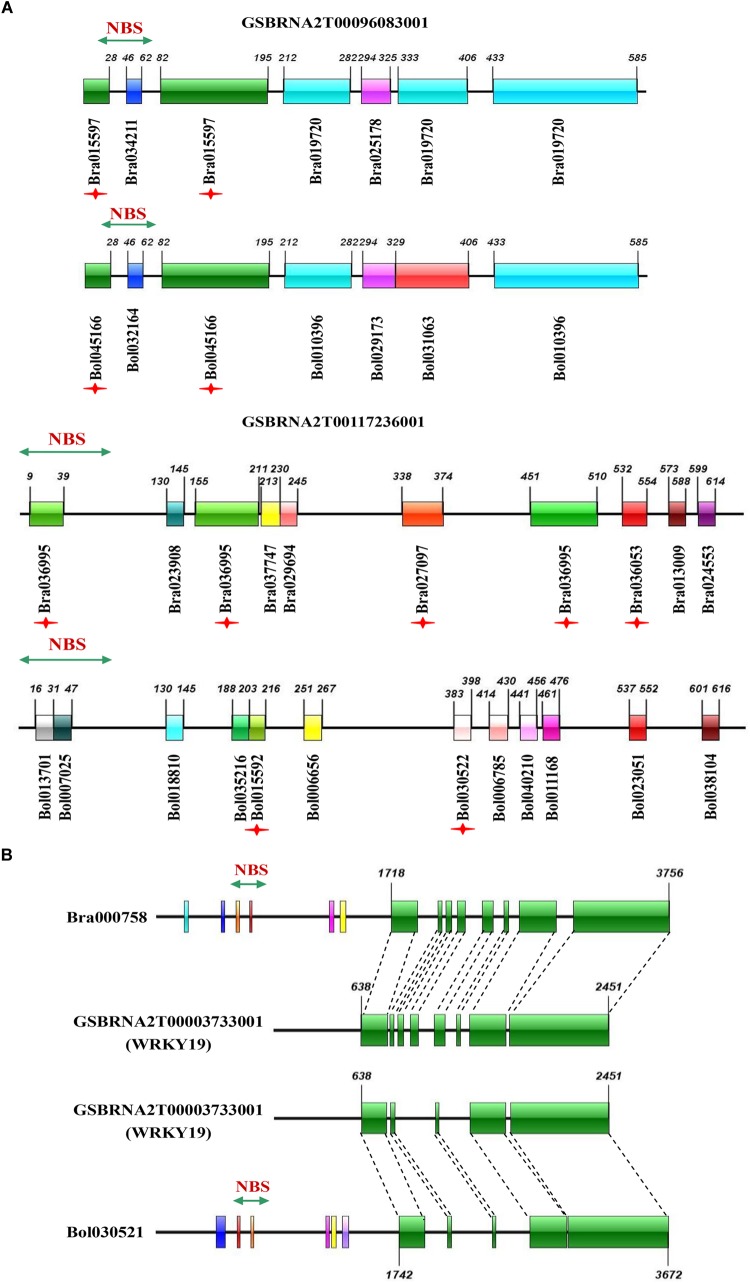
Sequence comparison of novel NBS-encoding genes in *B. napus* with the best matching genes in its progenitor species *B. rapa* and *B. oleracea*, and missing genes with the best matching genes in *B. napus*. **(A)** Sequence comparison of two novel genes in *B. napus* (*GSBRNA2T00096083001* and *GSBRNA2T00117236001*) with the best matching genes in *B. rapa* and *B. oleracea*. **(B)** Sequence comparison of two missing genes (*Bra000758* and *Bol030521*) with the best matching genes in *B. napus*. The colorful bars represent homologous blocks. The digits on the bars indicate the beginning and end of each homologous block.

The third group represented the birth and death of NBS-encoding genes in *B. napus* relative to its two progenitor species, including evolutionary pattern 5 from the results, where genes experienced large-scale sequence variation (effectively generating a novel gene), and evolutionary pattern 6 from the results, where genes from the progenitor species disappeared in *B. napus*. As an example of putative gene birth, two genes in the phylogenetic tree, GSBRNA2T00096083001 and GSBRNA2T00117236001, showed extreme sequence divergence from the other genes in the corresponding cluster. Sequence comparison of these two genes with the best matching genes in progenitor species revealed that these two genes combined sequences from several genes in the progenitor species, including NBS-encoding genes providing the NBS domains and non-NBS-encoding genes providing the other sequences, to generate new NBS-encoding genes in *B. napus* (Figure 6A). A putative mechanism for this is non-homologous recombination, either as a result of ancestral homoeology relationships (A-C or from triplicated regions within the A and C genomes; Parkin et al., 2014) or ectopically via the gene copies themselves as a substrate. Two genes (Bra000758 and Bol030521 from progenitor species), were also found to have been separated from their NBS-encoding domains in *B. napus*, indicating the “death” of these two genes in *B. napus*. Sequence comparison of these two genes with the best match genes in *B. napus* revealed that nearly half of the sequence of these two genes was retained (from approximately 1700 bp to the end of the gene), but that the NBS domain had disappeared (Figure 6B). These results suggest that both gene birth and death for NBS- encoding genes in *B. napus* are based on processes affecting original NBS-encoding genes, which are either subjected to large scale sequence variation to generate new genes or domain deletion resulting in gene death, with both processes putatively mediated by non-homologous recombination.

A caveat to our results is that single genome sequences of *B. rapa, B. oleracea*, and *B. napus* are being used to infer species and population-wide evolution patterns; a shortfall of many current genomic studies where multiple reference genomes are not yet available for individual species (Golicz et al., 2015). As the exact progenitor genotypes of *B. napus* are also unknown (Chalhoub et al., 2014), it is also difficult to predict with certainty which genomic variants are representative of evolutionary patterns post-allopolyploid formation, and which may have been inherited from different genotypes of the diploid progenitors than those used to create the genome references. This may also explain some of the results where the best gene match to NBS-encoding genes in *B. napus* was outside the *Brassica* genus: although horizontal gene transfer is a possibility, distinguishing this from incomplete lineage sorting with currently available genomic resources is challenging. Genome sequence quality can also play a major role: although the *Brassica* genome assemblies are predicted to cover most of the gene space (Wang et al., 2011;Chalhoub et al., 2014; Parkin et al., 2014), these genome references are still incomplete, which can significantly affect the numbers of genes detected. Nevertheless, our results predict some interesting patterns for resistance gene evolution in *B. napus*, forming a basis for further investigation pending additional genomic resources in this young allopolyploid crop.

#### More NBS-Encoding Genes Were Identified in *B. rapa* Than in Close Relative *B. oleracea*

Many more NBS-encoding genes were found in *B. rapa* than in *B. oleracea*. It has been proposed previously by molecular phylogenies that a clade containing *B. oleracea, B. rapa* and their respective wild relatives originated 6.47 Mya, late in the Miocene, and that finally *B. oleracea* varieties and their wild relatives diversified from this clade 1.44 Mya (Arias et al., 2014). The later diversification of *B. oleracea* relative to *B. rapa* might offer an explanation for why the C_o_ genome of *B. oleracea*, despite being significantly larger (630 Mb) than the A_r_ genome of *B. rapa* (485 Mb), contained fewer functional NBS-encoding genes. Additionally, *B. oleracea* varieties and their wild relatives were found to diversified in the northeastern Mediterranean and later spread through the rest of Europe (Sinskaia, 1928; Arias et al., 2014), while multiple divergence regions have been proposed for *B. rapa*, including Europe, central China, west Asia and north Africa (Zohary et al., 2012; Annisa et al., 2013; Guo et al., 2014). These multiple centers for divergence in *B. rapa* might provide another explanation for the greater prevalence of putatively functional NBS-genes in the A_r_-genome relative to the C_o_ genome: these may have evolved in response to the need to adapt to variable environments and disease pressures, and later spread between subpopulations. In future, with the availability of more, high-quality genome references, it may be interesting to compare NBS-encoding gene divergence between different genotypes and cultivars of *B. rapa* and *B. oleracea* (as well as *B. napus*), in order to fully elucidate the different evolutionary trajectories of resistance genes in response to genetic diversification, domestication and other genomic and environmental selective pressures.

#### A High Proportion of Mapped QTLs Against the Three Major Diseases of *B. napus* Were Repeatedly Identified and Showed Close Associations With Predicted NBS-Encoding Genes

We reviewed current studies covering QTL mapping of blackleg, clubroot, and *Sclerotinia* stem rot, the three major diseases of *B. napus*. We found that QTLs of these three diseases were unevenly distributed on chromosomes of *B. napus*, with the number of QTLs ranging from four to 14 (Table 5). This uneven distribution of QTLs across the chromosomes of *B. napus* was similar to the uneven distribution of NBS-encoding genes we observed. The three chromosomes with the most QTLs detected for resistance against these pathogens were A_n_09 with 14 QTLs, C_n_3 with 14 QTLs, and C_n_4 with 13 QTLs; this coincides with the finding that A_n_09 and C_n_3 were two of the three chromosomes with the most NBS-encoding genes (Figure 2). The similar distribution of disease resistance QTLs and NBS-encoding genes supports the close associations of NBS encoding genes with disease resistance against blackleg, clubroot and *Sclerotinia* stem rot in *B. napus*.

**Table 5:** The summary of mapped QTL against blackleg, clubroot and *Sclerotinia* stem rot in *B. Napus.*

Chromosome	Total QTL number	Blackleg		Clubroot		*Sclerotinia* stem rot	
				
		QTL	Repeatedly	QTL	Repeatedly	QTL	Repeatedly
		number	identified QTL^a^	number	identified QTL^a^	number	identified QTL^a^
A_n_01	11	8	6, 2	1	0	2	0
A_n_02	8	4	3	1	0	3	2
A_n_03	12	4	0	5	4	3	0
A_n_04	4	2	0	1	0	1	0
A_n_05	4	2	0	0	0	2	0
A_n_06	12	6	0	1	0	5	3
A_n_07	6	3	0	0	0	3	0
A_n_08	6	3	3	1	0	2	0
A_n_09	14	9	6, 3	0	0	5	2, 2
A_n_10	5	3	2	0	0	2	0
C_n_1	7	5	3	0	0	2	0
C_n_2	9	4	2	1	0	4	2
C_n_3	14	2	0	3	0	9	0
C_n_4	13	7	5	2	0	4	2
C_n_5	8	3	2	1	0	4	0
C_n_6	10	5	3	0	0	5	4
C_n_7	7	4	0	1	0	2	0
C_n_8	10	3	2	0	0	7	0
C_n_9	10	4	0	1	0	5	3
Total	170	81	42	19	4	70	20


*^*a*^The overlapping or neighboring located QTL (less than 1 Mb) were considered as repeatedly identified QTL. The digit indicated the QTL number that repeatedly identified in a locus.*

We also investigated overlapping and neighboring (within 1 Mb) QTL intervals to find repeatedly identified QTLs: approximately half (42/81) of resistance QTLs against blackleg were repeatedly identified. A total of 13 of these loci were identified for blackleg resistance (on A_n_01, A_n_02, A_n_08, A_n_09, A_n_10, C_n_1, C_n_2, C_n_4, C_n_5, C_n_6, and C_n_8). One locus on chromosome A_n_03 contained 4 of 19 resistance QTLs against clubroot, most likely corresponding to the major resistance gene *CRa* previously identified in this region (Ueno et al., 2012). Of the *Sclerotinia* stem rot resistance QTLs, 20 of 70 were repeatedly identified in 8 loci on A_n_02, A_n_06, A_n_09, C_n_2, C_n_4, C_n_6, and C_n_9 (Table 5 and Supplementary Table S2). Co-localized QTL detected across multiple studies are highly credible candidates for resistance against these three diseases. Across all QTL, 41.76% of intervals were co-located with NBS-encoding genes, which verified the close associations between the NBS-encoding genes and QTLs against these pathogens. Three NBS-encoding genes, (GSBRNA2T00081287001, GSBRNA2T00021121001, and GSBRNA2T00098068001) were located within overlapping QTLs against all three diseases, while 47 NBS-encoding genes were located within overlapping QTLs against two diseases. These results suggested that several common factors might exist for *B. napus* that confer resistance against clubroot, blackleg and *Sclerotinia* stem rot, and that these key factors may comprise NBS-encoding genes.

## Author Contributions

YF and DF designed the research. YF, YZ, BL, DZ, and HY analyzed the data. YF wrote the manuscript. AM assisted with interpretation of results, manuscript writing, and revision.

## Conflict of Interest Statement

The authors declare that the research was conducted in the absence of any commercial or financial relationships that could be construed as a potential conflict of interest.

## Footnotes

1 http://brassicadb.org/brad/

2 http://pfam.xfam.org/

3 http://pfam.janelia.org/

4 http://smart.embl-heidelberg.de/

5 http://embnet.vital-it.ch/software/COILS_form.html

6 http://brassicadb.org/brad/

7 http://visualization.ritchielab.psu.edu/phenograms/plot

8 http://tree.bio.ed.ac.uk/software/figtree/

9 http://brassicadb.org/brad/
