# Supplementary material for: NBS-Encoding Genes in Brassica napus Evolved Rapidly After Allopolyploidization and Co-localize With Known Disease Resistance Loci
Source: Front Plant Sci. 2019 Jan 30;10:26. doi: 10.3389/fpls.2019.00026 (PMC6363714; doi:10.3389/fpls.2019.00026)
Supplement: Supplementary file 1 [file Data_Sheet_1.doc]

NBS-encoding genes in *Brassica napus* evolved rapidly after allopolyploidization and co-localise with known disease resistance loci

Ying Fu^1^, Yaofeng Zhang^1^, Annaliese S. Mason^2^, Baogang Lin^1^, DongqingZhang^1^, Huasheng Yu^1^* and Donghui Fu^3^*

Figure S1. Phylogenetic tree of NBS-encoding genes in B. napus and progenitor species.

Figure S2. **Evolutionary patterns of NBS-encoding genes of *B. napus*.** a. NBS-encoding genes of *B. napus* that inherited from two progenitor species. b. NBS-encoding genes of *B. napus* that only inherited from single progenitor species. c. gene expanding events for NBS-encoding genes in *B. napus* relative to the progenitor species. d. the loss of NBS-encoding R-genes in *B. napus* from progenitor species. e. different divergence rates of NBS-encoding R-genes in *B. napus* relative to other homologous NBS-encoding R-genes.

Figure S2
