# Supplementary figures and images for: NBS-Encoding Genes in Brassica napus Evolved Rapidly After Allopolyploidization and Co-localize With Known Disease Resistance Loci
Source: Front Plant Sci. 2019 Jan 30;10:26. doi: 10.3389/fpls.2019.00026 (PMC6363714; doi:10.3389/fpls.2019.00026)

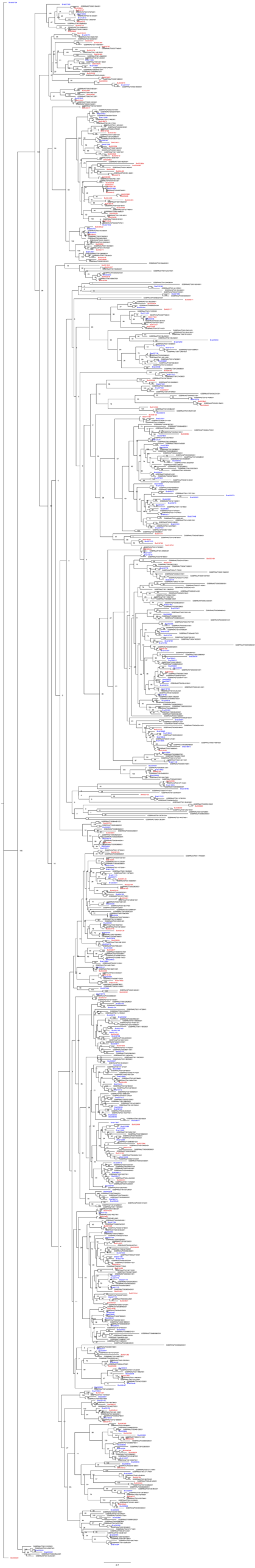

Supplement: Supplementary file 3 [file Image_1.PNG]
